# Periodontal probing on digital images compared to clinical measurements in periodontitis patients

**DOI:** 10.1038/s41598-021-04695-6

**Published:** 2022-01-31

**Authors:** Hye-Min Chung, Jin-Young Park, Kyung-A. Ko, Chang-Sung Kim, Seong-Ho Choi, Jung-Seok Lee

**Affiliations:** 1 Department of Periodontology, Research Institute for Periodontal Regeneration, Yonsei University College of Dentistry, 50 Yonsei-ro, Seodaemun-gu, Seoul, 03722 Republic of Korea; 2 Innovation Research and Support Center for Dental Science, Yonsei University Dental Hospital, Seoul, Republic of Korea

**Keywords:** Biological techniques, Anatomy, Diseases, Health care, Medical research

## Abstract

The aim of the study was to compare the supra-alveolar gingival dimension (GD) and the clinical pocket probing depth (PD) by combining data from an intraoral scanner (IOS) and cone-beam computed tomography (CBCT) and identify the clinical features affecting the clinical PD. 1,071 sites from 11 patients were selected for whom CBCT, IOS images, and periodontal charts were recorded at the same visit. CBCT and IOS data were superimposed. GD was measured on cross-sectional images of the probed sites. The level of agreement and correlation between GD and PD were assessed for the entire population and within groups (treated vs untreated, bleeding on probing [BOP] vs no BOP, and PDs of 0–3 mm vs 4–5 mm vs ≥ 6 mm). The mean [± SD] difference between GD and PD was 0.82 [± 0.69] mm, and they were positively correlated (r = 0.790, p < 0.001). The correlations between GD and PD were stronger for untreated sites, sites with BOP, and sites with a larger PD. Within the limitations of this study, the similarity between GD and PD may suggest a possible tendency of overestimation when recording PD.

## Introduction

Despite many advances in dental technology, the periodontal probe remains the most frequently used diagnostic tool for assessing the health status and attachment level of periodontal tissues^1^. It is well known that the accuracy and reproducibility of the clinical pocket probing depth (PD) can be affected by the probing force, presence of periodontitis, type of periodontal probe, and probing site^2–4^. The resulting errors can impact clinical decision-making, especially during longitudinal monitoring of the periodontal status^5^.

Moreover, clinical probing depth is one of the most frequently used parameters in the vast areas of clinical trials and case studies in the field of periodontology. However, the reliability of probing data is regarded to be low and more importantly the tendency of errors that occur during probing remains unclear^1,6^. Even though it is widely accepted that clinical probing is prone to errors due to numerous aforementioned clinical factors, it would be helpful to be knowledgeable about the predisposition of these errors when interpreting probing data^7^.

An intraoral scanner (IOS) is a device that directly captures images of the oral cavity. Numerous studies have shown that an IOS can be highly accurate^8–10^. Also in the field of clinical periodontology, our research team has recently conducted a mucogingival study comparing conventional probe data with digital data obtained using an IOS, and concluded that IOS data are more accurate and reproducible than using a periodontal probe to measure the width of keratinized gingiva^11^. Such an accurate representation of the gingival contours can be superimposed with cone-beam computed tomography (CBCT) data to determine the relationship between the gingiva and bone^12^.

It is well accepted that the gingival dimension (GD) comprises the 2-mm of supracrestal attached tissues and the 1-mm gingival sulcus^13^, whereas the normal probing sulcus depth is considered to range from 1 to 3 mm in healthy gingiva^14^. Due to the errors inherent to clinical probing and the unclear clinical presentation of the cementoenamel junction, clinical data are subject to inaccurate recordings of the clinical attachment loss and the severity of periodontal disease. Therefore, to appropriately interpret clinical probing data, they need to be clinically compared to the GD.

Based on the above-described situation, the null hypothesis of this study was that there will be no correlation between GD and PD. The aim of this study was to evaluate the tendency of clinical pocket probing depth by comparison with the supra-alveolar GD measured using IOS and CBCT data. Furthermore, the clinical features that affect clinical probing will be determined.

## Results

### Selected samples

This study analyzed 1071 sites from 179 teeth of 11 patients. For the group analyses, there were 300 untreated sites and 771 treated sites; 415 sites with BOP and 656 sites with no BOP; and 892 sites with a PD of 0–3 mm, 138 sites with a PD of 4–5 mm, and 41 sites with a PD of ≥ 6 mm (Table 1).

**Table 1:** Results showing the number of sites in each group, Pearson’s correlation coefficients and the mean differences between GD and PD.

	Sites (n)	r	p value	Mean [± SD]	p value
**Periodontal treatment**					
Treated	772	0.75	p < 0.001	0.83 [± 0.68]	p = 0.091
Non-treated	299	0.86	P < 0.001	0.76 [± 0.59]	
**Bleeding on probing site**					
(—)	415	0.69	p < 0.001	0.84 [± 0.66]	p = 0.072
( +)	656	0.85	p < 0.001	0.76 [± 0.64]	
**Clinical probing depth**					
0-3 mm	892	0.42	p < 0.001	0.87 [± 0.66]^a^	p < 0.001
4-5 mm	138	0.66	p < 0.001	0.51 [± 0.53]^b^	
≥ 6 mm	41	0.79	p < 0.001	0.47 [± 0.52]^b^	
Total	1071	0.79	p < 0.001	0.82 [± 0.69]	

r = Pearson’s correlation coefficient.

Bonferroni post t test, significant difference compared to 0–3 mm group (a > b, p < 0.001).

### Agreement and correlation between GD and PD

**Figure 1:**
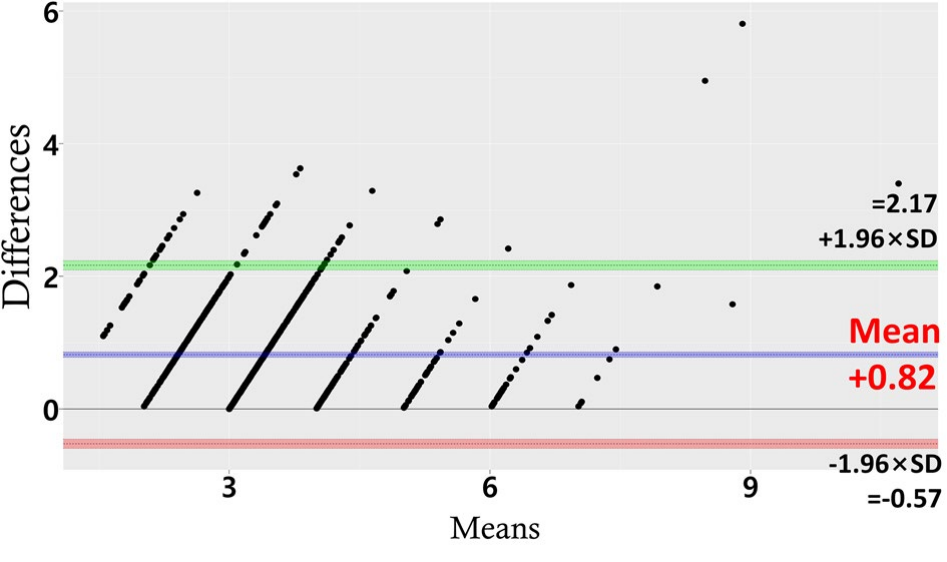
Bland–Altman plot of the mean of GD and probing depth (PD) (*x*-axis) versus the difference between GD and PD (*y*-axis). The mean difference (blue line) between GD and PD was 0.82 mm. The limits of agreement (green and red lines) are defined as the mean difference plus and minus 1.96 × the standard deviation. The coordinates never cross below 0 mm, because GD is always larger than or equal to PD. Since the lower limit is 0 mm, the two measurement methods had limits of agreement between 2.17 and 0 mm.

In the Bland–Altman plot (Fig. 1), the mean [± SD] difference between GD and PD was 0.82 [± 0.69] mm. In that figure, the coordinates never cross below 0 mm, because GD is always larger than or equal to PD. The limits of agreement were defined as the mean difference plus and minus 1.96 × the standard deviation. Even though the two measurement methods had limits of agreement between 2.17 and − 0.6 mm, the difference between GD and PD ranged between 0 and 2.17 mm. There was a significant positive Pearson’s coefficient for the correlation between GD and PD (r = 0.790, p < 0.001).

#### Treated versus untreated sites (Fig. 2A1,A2)

**Figure 2:**
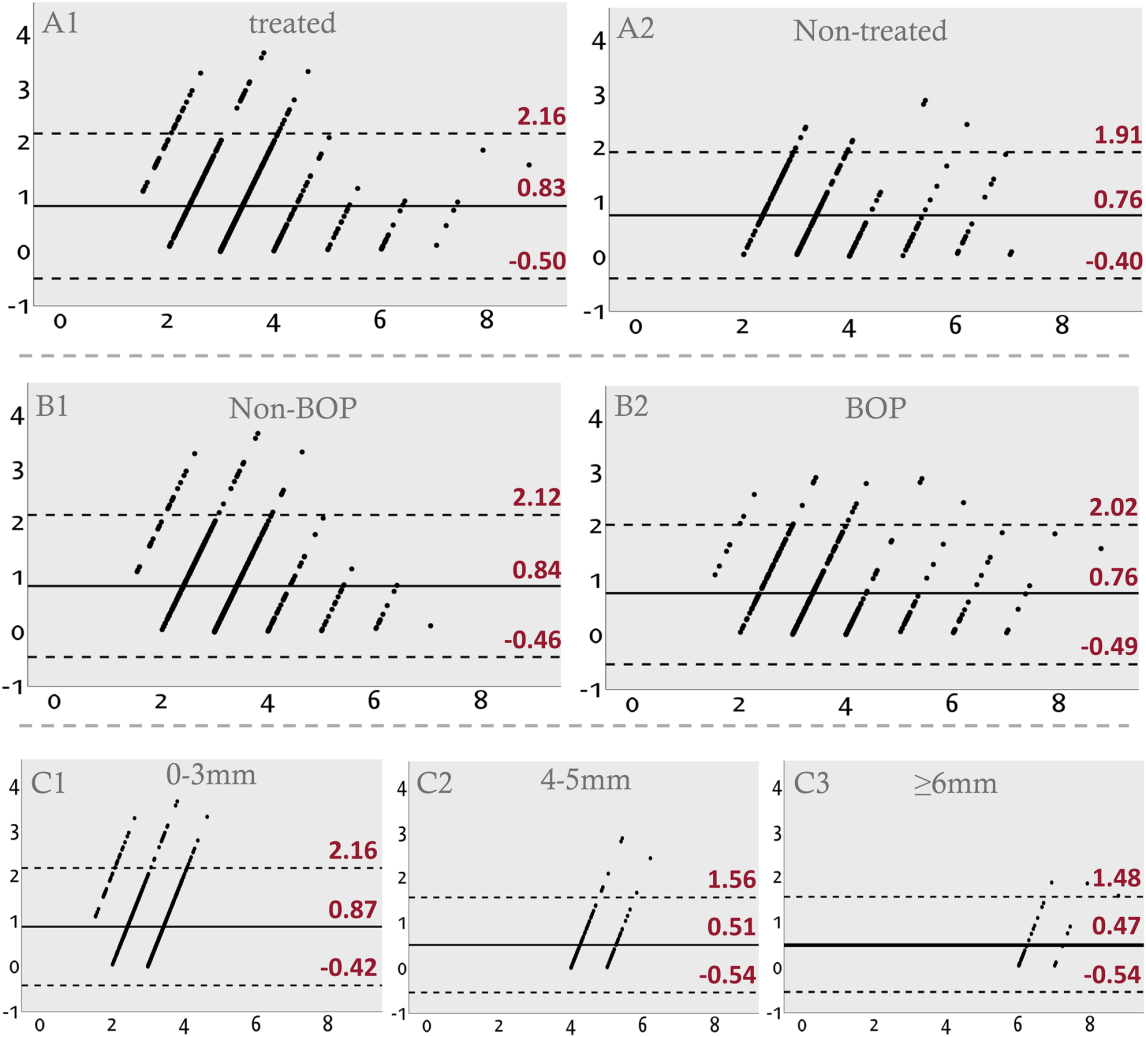
Bland–Altman plots for each group according to clinical features affecting the clinical PD. (A1) Sites treated for periodontitis. (A2) Sites with periodontitis that were not treated. (B1) Sites without BOP. (B2) Sites with BOP. (C1) Sites with a PD of 0–3 mm. (C2) Sites with a PD of 4–5 mm. (C3) Sites with a PD of ≥ 6 mm.

There was no statistically significant difference between untreated sites (0.76 [± 0.59] mm) and the treated sites (0.83 [± 0.68] mm) in terms of the mean [± SD] difference between GD and PD. Stronger correlation was found, however, at the untreated sites (r = 0.86, p < 0.001) compared to the treated sites (r = 0.75, p < 0.001).

#### BOP versus no-BOP sites (Fig. 2B1,B2)

There was no statistically significant difference between the sites with BOP (0.76 [± 0.69] mm) and those without BOP (0.80 [± 0.66] mm) in terms of the mean [± SD] difference between GD and PD. Stronger correlation was found, however, at sites with BOP (r = 0.852, p < 0.001) than at those without BOP (r = 0.691, p < 0.001).

#### PDs of 0–3 mm versus 4–5 mm versus ≥ 6 mm (Fig. 2C1–C3)

The mean [± SD] difference between GD and PD was largest (0.87 [± 0.66] mm) for a PD of 0–3 mm and smallest for a PD of ≥ 6 mm (0.47 [± 0.52] mm). There were significant differences between the 0- to 3-mm group and the other groups (p < 0.001), but not between the 4- to 5-mm group and the ≥ 6-mm group (p = 1.00). Pearson’s coefficients showed that the correlation between GD and PD became stronger as PD increased (r = 0.42, 0.66, and 0.79 for the 0–3, 4–5, and ≥ 6 mm groups, respectively; p < 0.001).

## Discussion

The current study is the first to compare digitized GD and PD values using a comprehensive set of clinical data including full periodontal chart, intraoral scan and CBCT from the same visit. This study was designed based on the concept that the difference between PD and GD would produce a value that represents the supracrestal tissue attachment (previously known as “biologic width”), which is known to be approximately 2 mm. The main findings of this study were (1) the mean difference between GD and PD was smaller than 2 mm, (2) the difference became smaller at deeper periodontal pockets, and (3) there was a moderately strong positive correlation between the two methods. Therefore, the null hypothesis that there will be no correlation between GD and PD was rejected.

In this study, the mean difference between GD and PD was 0.82 mm, which is smaller than the widely accepted width of supracrestal attached tissue of around 2 mm as found by Gargiulo et al. in a histologic study of cadavers^13^. Although there will be some intra- and interindividual variability in measurements of the supracrestal attached tissue, the value obtained in this study is smaller than that found in a meta-analysis (2.15–2.30 mm)^15^. One obvious possible explanation is the current study involving patients diagnosed with severe generalized periodontitis. In the presence of inflammation, the probe tip will penetrate the junctional epithelium and stop at the most-coronal part of the noninflamed connective-tissue ligament^16–18^. However, similar results were obtained even at healthy sites with no BOP and a PD of 0–3 mm. Other clinical studies using transgingival probing have also found similarly small width of supracrestal attached tissues^14,15^. The most likely explanation for this discrepancy would be the rounding error when using a periodontal probe: PDs are often rounded off by the examiner to the nearest millimeter mark on the periodontal probe, thereby introducing subjective bias. Previous studies of the pocket PD and keratinized gingiva width measurements using a periodontal probe have shown that probing values were often overestimated by 1 mm^11,14^. The results of the present study also suggest that examiner bias in pocket probing leads to overestimation.

A Bland–Altman plot includes 95% limits of agreement to facilitate visual judgements about how well two methods of measurement agree^19^. A smaller range between these two limits indicates better agreement. The limits of agreement in the present study ranged from − 0.56 to 2.17 mm, however, since the coordinates never crossed below zero, the actual range of difference can be said to be between 0 and 2.17 mm. This is a reasonable range considering that the upper limit coincides with the width of supracrestal attached tissue. The difference between GD and PD will be closer to the upper limit when the periodontal health is good, whereas it will be nearer to 0 mm in the presence of inflammation. This was shown clearly by the group analyses performed in this study, in which the correlation between GD and PD was stronger at untreated sites, in the presence of BOP, and when PD was larger than 4 mm. These findings indicate that there was a strong overall correlation between GD and PD. Based on the limits of agreement together with the mean difference proposed by the current study, the digital technique using CBCT and IOS data appears to represent GD in reasonable degree of accuracy and correlation with PD. With further studies in larger population to confirm that PD can be accurately represented using the digitized GD, this method could potentially be utilized in future practice for predicting the pocket PD and monitoring periodontal health.

One of the limitations of this study was the lack of histologic analysis, which would provide the gold standard representation of PD and GD. Therefore, the accuracy of the measurements performed on CBCT and IOS images needs to be clarified using references in the literature. CBCT-based linear measurements of oral structures have been previously confirmed as being highly accurate and reproducible, with a mean measurement error of less than 0.01 mm^20^. A study of the accuracy of the IOS used in the current study revealed high trueness and precision with very small margins of error between 0.01 and 0.03 mm^8^. Nevertheless, these devices can still have flaws. CBCT can be erroneous as a result of patient movement during scan. Also, CBCT images can be disrupted by metal artifacts, which can cause difficulty during GD measurements. Another clear limitation in this study was the possible disparity between the measurement site of GD and PD. Due to the retrospective design of this study, PD investigators could not be calibrated for the exact location of probe insertion. In addition, this study was performed exclusively on patients who were diagnosed with severe generalized periodontitis. Therefore, the results obtained from this study needs to be interpreted with caution. A further study is needed including periodontally healthy subjects to represent the general population.

One may raise the concern regarding the paucity of data in this study due to the small number of patients that have been included. However, site-wise analyses were performed rather than patient-wise analysis to compare GD with PD, and the number of sites were sufficient to fulfill that objective. Previous clinical studies on probing accuracy have included many patients but only using single-tooth site per patient. In addition, pocket probing is strictly a mechanical measurement procedure, in which broader patient level factors would sustain little or no influence on the outcome.

Despite the accuracy of the two digital methods, they have rarely been applied in the periodontal field for diagnostic purposes due to the associated radiation exposure and the low cost-effectiveness of the procedure given that probing is merely a screening tool for disease detection^21^. Nonetheless, digital techniques are being incorporated increasingly frequently in both research and practice for various treatments^22^. It is useful for clinicians to be aware of the relationship between the digitally measured GD and the clinical PD, so that digital measurements can be utilized in the absence of probing data. The data obtained in the present study can be useful for medically compromised patients in whom probing may be contraindicated^23,24^.

To conclude, within the limitations of this study, the similarity between the GD and PD measurements may suggest a possible tendency of overestimation when recording PD. The outcome from this study can be meaningful since success or failure of periodontal treatments can often be determined by small gains or losses of clinical attachment levels. Correct documentation of probing data along with the knowledge of the tendency of errors can be valuable for long term monitoring of periodontal status. Since the digitized GD measurements showed consistent correlation with clinical probing, this data can be utilized in further studies for future clinical application.

## Methods

### Study design

This study retrospectively analyzed clinical data from patients who visited Yonsei University Dental Hospital for the treatment of periodontal disease from 2018 to 2020. All included patients had generalized (> 30% of teeth involved) severe periodontal inflammation as well as > 50% of alveolar bone loss along with the dental root in radiography, with clinical features of a PD of > 7 mm, clinical attachment loss of > 5 mm, and easy bleeding on probing (BOP). Based on the consensus report of the 2017 World Workshop on the periodontal classification, those patients were diagnosed as generalized periodontitis of stages III and IV with grade C (rapid progression). This study was approved by the Yonsei University Dental Hospital, Institutional Review Board (2-2020-0086), and was conducted in accordance with the Helsinki Declaration. The Institutional Review Board of Yonsei University Dental Hospital abides with Good Clinical Practice and the regulatory requirements, and it has been approved that informed consent can be waivered for this retrospective study.

### Sample

The study sample consisted of 11 patients who fulfilled the following eligibility criteria:

1. Diagnosed with or who had received treatment for generalized severe periodontitis.
2. Availability of all three clinical data sets of CBCT, full mouth IOS images, and periodontal charts recorded at the same visit.
3. Full periodontal probing charts including for BOP performed at six sites per tooth.

### Data acquisition

**Figure 3:**
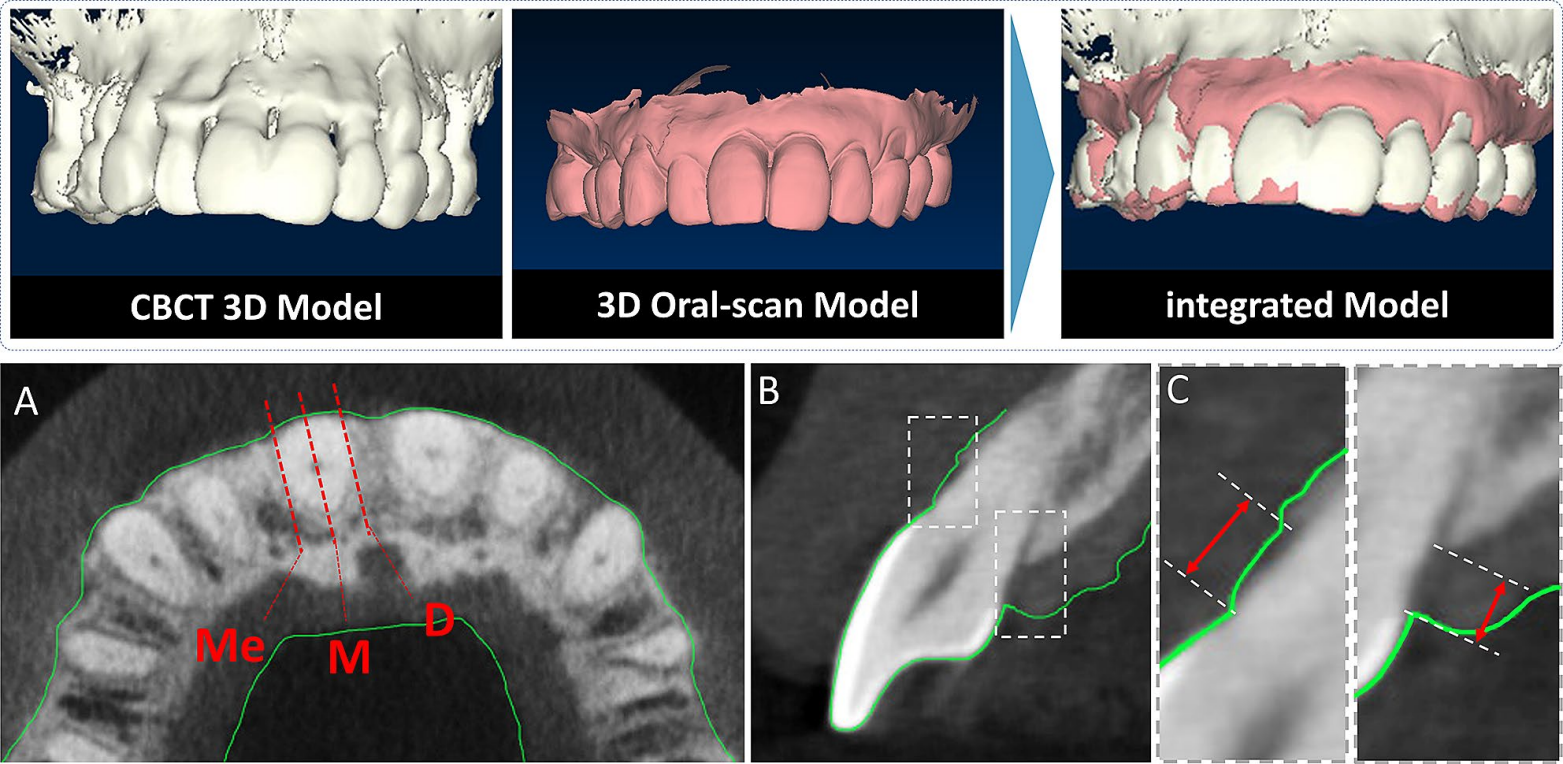
Cone-beam computed tomography (CBCT) images were acquired and integrated with an oral-scan model using 3D reconstruction software (OnDemand3D; Cybermed, Seoul, South Korea, https://www.ondemand3d.com/). The supra-alveolar gingival dimension (GD) was measured. (**A**) Axial image showing the positions selected for vertical cross-sectioning (mesial, middle, and distal). (**B**) Cross-sectional image of the dentogingival unit for GD measurement. (**C**) GD was defined as the distance between the most-coronal point of the marginal gingiva and the underlying alveolar bone.

All clinical PDs were measured using a color-coded periodontal probe (UNC-15). At the patient visit to the clinic, full mouth periodontal pocket probing was performed by a periodontics resident or a dental professional. Probe tips were inserted parallel to the long axis of the tooth with a probing force of 25 N at six sites per tooth (mesiobuccal, midbuccal, distobuccal, mesiolingual, midlingual, and distolingual). BOP was present if bleeding from the probed site was detected shortly after probing, which was recorded on the periodontal chart as positive or negative. An intraoral scan was performed using an oral scanner (Trios 3; 3Shape, Copenhagen, Denmark) at the same visit as the periodontal probing. Any blood or saliva that remained on the gingival margin from the full mouth probing was cleaned and dried using a 3 in 1 syringe, and then an oral scan was performed of the entire dentition and the gingiva up to the mucogingival junction. CBCT (Alphard 3030 device; Asahi Roentgen, Tokyo, Japan) was also performed on the same visit with the purpose of diagnostic imaging for dental implant therapy at an edentulous site. The CBCT settings were as follows: tube voltage, 80 kVp; tube current, 8 mA; exposure time, 17 s; field of view, 102 mm × 102 mm; and voxel size, 0.2 mm. CBCT data was saved in the DICOM format, and intraoral scan data in the STL file format. CBCT and IOS data were imported into a 3D reconstruction software (OnDemand3D; Cybermed, Seoul, South Korea, https://www.ondemand3d.com/) and superimposed based on three teeth as reference points. The supra-alveolar GD was defined as the distance between the most-coronal point of the marginal gingiva and the underlying alveolar bone in the cross-sectional image obtained in the apicocoronal axis of the tooth (Fig. 3). Cross-sectional images were obtained from six sites of each tooth representing the probed sites (mesiobuccal, midbuccal, distobuccal, mesiolingual, midlingual, and distolingual), and GD was measured on each cross-section using the measurement tool on the software. All measurements were made by the same trained examiner (H.M.C.) and were repeated twice with a 2-week interval (intraexaminer correlation coefficient = 0.89, confidence interval = 0.88–0.91).

### Outcome

The main outcome of the study was the level of agreement between GD and PD as quantified by the mean difference between the two methods. The coefficient for the correlation between GD and PD was also calculated to evaluate the strength of the association. The sites were analyzed for the entire sites and then divided into groups for evaluations according to the following three clinical features as potential modifying factors, which were the secondary outcomes:

i. Treated versus untreated sites for periodontitis.
ii. Sites with BOP versus sites without BOP.
iii. Sites with PDs of 0–3 mm versus 4–5 mm versus ≥ 6 mm.

### Statistical analysis

The Bland–Altman method was used to analyze the degree of agreement between GD and PD. Pearson’s correlation coefficient (r) was used to analyze the correlation between the GD and PD. Mean differences between two groups were compared using the unpaired *t*-test, while those between three groups were compared using one-way ANOVA and the Bonferroni correction. Values of *r* were statistically compared between groups with a 95% confidence level using a dedicated statistical software (SPSS version 22.0; IBM, Armonk, NY, USA, https://www.ibm.com/analytics/spss-trials).

### Sample size calculation

Sample size was calculated based on a previous study, in which measurements of clinical GD and PD were conducted to analyze the width of supracrestal attached tissue^14^. A statistical software (Medcalc version 20.011; Ostend, Belgium, https://www.medcalc.org/) was used that can perform sample size calculations for method-comparison studies based on the Bland Altman plot. Type I and II errors were set as 0.05 and 0.20, respectively. According to the previous study, the expected mean [± SD] of the difference between GD and PD was 1.13 [± 0.28], and the maximum allowed difference between methods was set as 2.30, as indicated by a systematic review on the width of supracrestal attached tissue^15^. This resulted in the minimum required number of nine pairs.

## Data Availability

The datasets generated during the current study are available from the corresponding author on reasonable request.
